# Genome-wide analysis of the *NAAT*, *DMAS*, *TOM*, and *ENA* gene families in maize suggests their roles in mediating iron homeostasis

**DOI:** 10.1186/s12870-021-03422-7

**Published:** 2022-01-17

**Authors:** Xin Zhang, Ke Xiao, Suzhen Li, Jie Li, Jiaxing Huang, Rumei Chen, Sen Pang, Xiaojin Zhou

**Affiliations:** 1 Biotechnology Research Institute, Chinese Academy of Agricultural Sciences, Beijing, 100081 China; 2 Department of Applied Chemistry, College of Science, China Agricultural University, Beijing, 100193 China; 3 Center for Biological Science and Technology, Institute of Natural Science, Beijing Normal University, Zhuhai, 519087 China; 4 College of Life Sciences, Beijing Normal University, Beijing, 100875 China

**Keywords:** Iron homeostasis, Nicotianamine aminotransferase, 2′-deoxymugineic acid synthase, Transporter of MAs, Efflux transporter of NA, Expression profile, Subcellular localization, Maize, Phytosiderophores

## Abstract

**Background:**

Nicotianamine (NA), 2′-deoxymugineic acid (DMA), and mugineic acid (MA) are chelators required for iron uptake and transport in plants. Nicotianamine aminotransferase (NAAT), 2′-deoxymugineic acid synthase (DMAS), transporter of MAs (TOM), and efflux transporter of NA (ENA) are involved in iron uptake and transport in rice (*Oryza sativa*), wheat (*Triticum aestivum*), and barley (*Hordeum vulgare*); however, these families have not been fully identified and comprehensively analyzed in maize (*Zea mays* L.).

**Results:**

Here, we identified 5 *ZmNAAT*, 9 *ZmDMAS*, 11 *ZmTOM*, and 2 *ZmENA* genes by genome mining. RNA-sequencing and quantitative real-time PCR analysis revealed that these genes are expressed in various tissues and respond differently to high and low iron conditions. In particular, iron deficiency stimulated the expression of *ZmDMAS1*, *ZmTOM1*, *ZmTOM3*, and *ZmENA1*. Furthermore, we determined protein subcellular localization by transient expression of green fluorescent protein fusions in maize mesophyll protoplasts. ZmNAAT1, ZmNAAT-L4, ZmDMAS1, and ZmDMAS-L1 localized in the cytoplasm, whereas ZmTOMs and ZmENAs targeted to plasma and tonoplast membranes, endomembranes, and vesicles.

**Conclusions:**

Our results suggest that the different gene expression profiles and subcellular localizations of ZmNAAT, ZmDMAS, ZmTOM, and ZmENA family members may enable specific regulation of phytosiderophore metabolism in different tissues and under different external conditions, shedding light on iron homeostasis in maize and providing candidate genes for breeding iron-rich maize varieties.

**Supplementary Information:**

The online version contains supplementary material available at 10.1186/s12870-021-03422-7.

## Background

Iron (Fe), an essential micronutrient for all organisms, acts as a cofactor for many enzymes and plays an important role in numerous cellular functions in plants, including respiration, photosynthesis, and chlorophyll biosynthesis. Iron deficiency in the human diet leads to health problems such as anemia, which affects more than one billion people. In plants, iron deficiency causes leaf senescence, which limits plant growth and reduces quality and yield [1]. However, plant cells must carefully modulate iron levels, as excessive intracellular iron is toxic. Therefore, plants have evolved complex mechanisms to balance iron acquisition, transport, storage, and detoxification [2–4].

Although most soils have relatively high levels of iron, iron has low solubility under aerobic conditions and therefore is often unavailable for plant uptake [5]. Plants have two strategies for iron acquisition [2]. Non-graminaceous plants use strategy I, which involves the reduction of ferric (Fe^3+^) to ferrous (Fe^2+^) iron on the root surface, and transport of Fe^2+^ into the root by the plasma membrane protein IRON-REGULATED TRANSPORTER1 (IRT1) [6, 7]. Strategy II occurs predominately in graminaceous plants and involves the biosynthesis of phytosiderophores (organic substances released by the roots that chelate iron), followed by the uptake of chelated iron. For example, mugineic acids (MAs) are secreted to the rhizosphere by transporters of MAs (TOM) [8]. Fe^3+^–MA complexes form in the rhizosphere and are transported into plant root cells by YELLOW STRIPE1/YELLOW STRIPE1–LIKE (YS1/YSL) iron transporters [9]. The MA biosynthetic pathway is conserved in graminaceous plants and uses nicotianamine (NA) as a precursor. NA is biosynthesized from three molecules of S-adenosyl-L-methionine by nicotianamine synthase (NAS). Then, NA is converted to deoxymugineic acid (DMA) by nicotianamine aminotransferase (NAAT) and 2′-deoxymugineic acid synthase (DMAS) [10, 11]. Although DMA is the final product of phytosiderophore biosynthesis in rice (*Oryza sativa*), it can be further converted into MA by a hydroxylation reaction in barley (*Hordeum vulgare*) and other graminaceous plants [12]. Although non-graminaceous plants do not produce DMA and MAs, they still produce NA to chelate Fe^2+^ and assist in iron trafficking [2, 13].

In addition to acting in iron uptake, phytosiderophores also participate in iron transport within the plant. Iron is transported in a chelated form in plants due to its low solubility and high reactivity. The iron chelators DMA, NA, and citrate assist in long-distance trafficking of iron in various species [14–16]. Since DMA–Fe^3+^ and NA–Fe^2+^ have been detected in the phloem sap, they are proposed to be chelators for phloem iron transport [17, 18]. OsYSL2, OsYSL9, and OsYSL15 are iron transporters in phloem with different selectivity for NA–Fe^2+^ or DMA–Fe^3+^, indicating possible switching of chelators between NA and DMA in phloem [19–21]. Citrate plays a major role in xylem iron transport, and citrate–Fe^3+^ and citrate–Fe^2+^ were detected in xylem sap of *Arabidopsis thaliana* and rice. Moreover, knockout of a citrate efflux transporter, RICE FERRIC REDUCTASE DEFECTIVE3-LIKE1 (OSFRDL1), leads to decreased citrate and iron contents in the xylem sap [14, 22]. Since DMA and NA concentrations in the xylem are significantly lower than that of citrate, they are considered to play minor roles in xylem iron transport. However, in response to iron deficiency, DMA accumulates in rice xylem sap, and the expression of the DMA efflux transporters *OsTOM1* and *OsTOM2* was induced in the root stele, indicating that DMA serves as a compensatory chelator in xylem iron transport in response to fluctuating iron status [23, 24].

Interfering with phytosiderophore biosynthesis and release generates strong iron-deficient phenotypes and growth defects. Two classical maize (*Zea mays* L.) chlorosis mutants, *ys1* and *ys3*, are characterized by a yellow stripe phenotype, resulting from impaired MA–Fe^3+^ uptake and disrupted phytosiderophore secretion, respectively [25, 26]. Typical iron deficiency also results from disturbed DMA/NA metabolism. Constitutive expression of *HvNAAT* in transgenic tobacco (*Nicotiana tabacum*) plants leads to overconsumption of NA, causing interveinal chlorosis in young leaves [27]. These observations imply that regulation of NA-MA metabolism is crucial for iron homeostasis in graminaceous plants. Furthermore, different DMA/NA ratios affected zinc (Zn) and iron contents in the embryo and endosperm of rice and altered the local distribution patterns of iron in the embryo [28], shedding light on how the balance between DMA and NA affects iron homeostasis.

In addition to providing precursors for DMA biosynthesis, cytosolic NA is also consumed by EFFLUX TRANSPORTER OF NA (ENA), which may export NA out of cells and transport NA between intracellular compartments [29]. Therefore, NA may act as a hub for phytosiderophore metabolism because it not only serves as the biosynthetic intermediate of phytosiderophores but also regulates metal transport. However, despite progress in functional characterization of individual NAAT, DMAS, TOM, and ENA members in rice and barley, many gaps remain in the understanding of their regulatory mechanisms in response to fluctuating environmental iron status. The expression of *NAAT* and *DMAS* genes is induced in roots under iron deficiency, indicating that accumulation of DMA is necessary to manage iron-deficient conditions [10, 30]. The release of NA and DMA/MAs is also regulated by different efflux transporters, as *OsTOM1* expressed in root cells is involved in the secretion of MAs to the rhizosphere, while *OsTOM2* and *OsTOM3* show specific expression patterns associated with iron transport in a few tissues [24]. The expression of *VACUOLAR MUGINEIC ACID TRANSPORTER* (*OsVMT*), a homolog of *OsTOM1*, was induced in roots under iron deficiency [31]. These observations suggest different expression specificities, subcellular localizations, and possible enzyme activities of NAAT, DMAS, TOM, and ENA members, which provide various levels of regulation in the production and secretion of DMA/MAs.

Maize is a major crop worldwide. Although the iron content of corn kernels is higher than that of brown rice [31], it is still insufficient to meet the nutritional demands of a growing human population. Therefore, breeding maize varieties with enriched iron content is essential, and elucidation of the NA-MA metabolic pathway may provide key information and gene resources. We previously reported the duplication of *NAS* family genes in maize, which suggests that the regulation of NA biosynthesis is finely tuned by multiple genes that have diverged in expression pattern or protein subcellular localizations [32]. This raises the question of whether NAAT, DMAS, TOM, and ENA members are also encoded by multi-gene families, which might enable the regulation of DMA biosynthesis and NA/DMA export in response to changing iron uptake, transport, detoxication, and storage. In this study, we explored genes encoding NAAT, DMAS, TOM, and ENA in the maize genome. Additionally, we analyzed their subcellular localization and expression in different tissues as well as in response to fluctuating environmental iron conditions. Our results provide a better understanding of the regulation of NA-MA metabolism and suggest gene resources for breeding iron-rich maize varieties.

## Results

### Genome-wide identification of genes associated with phytosiderophore biosynthesis and secretion in maize

To identify enzymes involved in the biosynthesis of phytosiderophores, as well as transporters for NA and MAs, we scanned the maize B73 genome using TBLASTN, BLASTP, and the Hidden Markov Model algorithm (HMMER) with previously identified NAAT, DMAS, TOM, and ENA proteins as queries (Table S1). Consistent with the observation that *ZmNAS* genes are encoded by a multi-gene family in maize, we found 5, 9, 11, and 2 genes encoding putative ZmNAAT, ZmDMAS, ZmTOM, and ZmENA proteins, respectively. Compared to genes of these families identified in wheat (*Triticum aestivum*), barley, and rice, maize tends to have a larger number of DMAS and TOM proteins (Table S2). The gene ID, chromosomal location, amino acid (aa) length, molecular weight, isoelectric point, and subcellular localization prediction of identified genes is shown in Table 1.

**Table 1:** Maize *ZmNAAT*, *ZmDMAS*, *ZmTOM*, and *ZmENA* genes

Gene name	Gene ID	Chromosome location (bp)	Protein length (aa)	cDNA length (bp)	Subcellular location	pI/MW
*ZmNAAT1*^*a*^	*Zm00001d053281*	Chr 4: 223,892,837-223,896,295	434	1543	cyto: 9, cysk: 2, chlo: 1, plas: 1, vacu: 1	6.51 / 47,386.64
*ZmNAAT-L1*	*Zm00001d007462*	Chr 2: 232,247,283-232,255,516	474	1685	chlo: 4, plas: 3, E.R.: 3, nucl: 2, cyto: 1, vacu: 1	5.66 / 51,515.18
*ZmNAAT-L2*	*Zm00001d048736*	Chr 4: 4,607,651–4,610,357	455	1771	cyto: 8, nucl: 3, plas: 2, mito: 1	7.97 / 48,446.75
*ZmNAAT-L3*	*Zm00001d053107*	Chr 4: 214,129,873-214,132,827	438	1694	cyto: 10, chlo: 2, pero: 2	5.77 / 47,806.41
*ZmNAAT-L4*	*Zm00001d016441*	Chr 5: 162,429,370-162,433,900	440	1792	cyto: 5, cysk: 4, nucl: 2, chlo: 1, pero: 1, E.R._vacu: 1	6.68 / 47,869.22
*ZmDMAS1*^*a*^	*Zm00001d028360*	Chr 1: 32,049,647-32,052,463	314	1209	chlo: 6, cyto: 6, mito: 2	7.58 / 35,377.61
*ZmDMAS-L1*	*Zm00001d003524*	Chr 2: 46,658,256-46,660,165	329	1310	chlo: 8, cyto: 4, mito: 2	6.02 / 36,106.36
*ZmDMAS-L2*	*Zm00001d003525*	Chr 2: 46,712,428-46,719,096	360	4502	chlo: 10, cyto: 2, nucl: 1, mito: 1	7.14 / 40,464.85
*ZmDMAS-L3*	*Zm00001d005932*	Chr 2: 193,258,782-193,262,494	343	1393	chlo: 6, cyto: 6, pero: 2	6.97 / 38,422.36
*ZmDMAS-L4*	*Zm00001d042869*	Chr 3: 182,508,943-182,514,234	310	1470	chlo: 10, mito: 4	6.21 / 34,561.36
*ZmDMAS-L5*	*Zm00001d000060*	Chr 10: 292,300-293,833	358	1339	chlo: 7, cyto: 3, mito: 2, extr: 2	5.68 / 39,022.09
*ZmDMAS-L6*	*Zm00001d025057*	Chr 10: 102,026,193-102,028,561	313	1465	cyto: 9, extr: 5	5.79 / 33,871.83
*ZmDMAS-L7*	*Zm00001d025528*	Chr 10: 121,509,129-121,526,342	344	3967	mito: 8.5, chlo_mito: 7.5, chlo: 5.5	8.59 / 38,278.28
*ZmDMAS-L8*	*Zm00001d025533*	Chr 10: 121,567,456-121,569,106	331	1350	cyto: 9, chlo: 2, mito: 2, pero: 1	5.82 / 36,556.76
*ZmTOM1*^*a*^	*Zm00001d041111*	Chr 3: 97,974,955-97,982,185	476	1780	plas: 9, vacu: 3, E.R.: 2	9.09 / 51,809.84
*ZmTOM2*^*a*^	*Zm00001d052435*	Chr 4: 189,981,671-189,987,040	589	2560	plas: 8, E.R.: 2, golg: 2, cyto: 1, vacu: 1	7.05 / 64,209.85
*ZmTOM3*^*a*^	*Zm00001d005001*	Chr 2: 153,144,332-153,148,743	492	2001	plas: 10, nucl: 1, cyto: 1, vacu: 1, E.R.: 1	6.56 / 54,209.15
*ZmTOM-L1*	*Zm00001d031789*	Chr 1: 201,868,285-201,873,117	503	1903	plas: 11, nucl: 1, vacu: 1, E.R.: 1	7.47 / 56,243.48
*ZmTOM-L2*	*Zm00001d005002*	Chr 2: 153,238,644-153,242,689	502	1917	plas: 8, vacu: 3, golg: 2, E.R.: 1	8.53 / 54,794.86
*ZmTOM-L3*	*Zm00001d040422*	Chr 3: 42,672,281–42,680,006	541	2344	plas: 13, vacu: 1	8.54 / 59,214.48
*ZmTOM-L4*	*Zm00001d040468*	Chr 3: 45,120,037-45,134,869	473	1708	plas: 6, vacu: 6, cyto: 1, E.R.: 1	8.78 / 51,693.82
*ZmTOM-L5*	*Zm00001d040947*	Chr 3: 81,710,564-81,722,446	317	1900	plas: 5, chlo: 4, mito: 2, E.R.: 2, pero: 1	9.46 / 34,997.54
*ZmTOM-L6*	*Zm00001d044640*	Chr 3: 233,816,809-233,822,007	494	1796	vacu: 8, plas: 4, E.R.: 2	9.04 / 54,178.57
*ZmTOM-L7*	*Zm00001d052434*	Chr 4: 189,873,712-189,877,696	508	1988	plas: 9, vacu: 2, golg: 2, E.R.: 1	8.93 / 55,000.13
*ZmTOM-L8*	*Zm00001d008227*	Chr 8: 1,969,000-1,975,048	480	1761	plas: 8, vacu: 4, cyto: 1, golg: 1	8.51 / 52,546.85
*ZmENA1*	*Zm00001d052532*	Chr 4: 192,393,400-192,396,824	452	1615	plas: 8, vacu: 4, cyto: 1, golg: 1	8.06 / 48,997.77
*ZmENA2*	*Zm00001d014611*	Chr 5: 55,313,125-55,314,896	188	714	plas: 6.5, E.R.: 4, cyto_plas: 4, vacu: 2, chlo: 1	6.30 / 20,095.53

Chromosome (Chr), amino acid (aa), coding DNA (cDNA), theoretical isoelectric point (pI), molecular weight (MW); The subcellular location of ZmNAAT, ZmDMAS, ZmTOM, and ZmENA proteins was predicted using WoLF PSORT (https://wolfpsort.hgc.jp/), cytosol (cyto), cytoskeleton (cysk), chloroplast (chlo), plasma membrane (Plas), vacuole (vacu), endoplasmic reticulum (E.R.), nucleus (nucl), mitochondria (mito), peroxisome (pero), extracellular (extr), Golgi apparatus (golg); ^a^ indicates that these genes were identified previously

Previously identified genes were annotated, and newly identified genes were named *ZmNAAT-like1–4*, *ZmDMAS*-*like1*–*8*, *ZmTOM*-*like1*–*8*, and *ZmENA1*–*2* according to their chromosomal locations. The amino acid lengths of the ZmNAATs and ZmDMASs varied modestly, ranging between 434–474 aa and 310–360 aa, respectively. TOM and ENA proteins belong to the major facilitator superfamily (MFS), but they show large variation in protein lengths, ranging between 317–589 aa and 188–452 aa, respectively, suggesting that TOMs and ENAs may vary in substrate affinity or subcellular localization. Most NAATs and DMASs were predicted to localize in the cytosol, but most TOMs and ENAs were predicted to localize in the plasma membrane or vacuolar membrane.

**Fig. 1:**
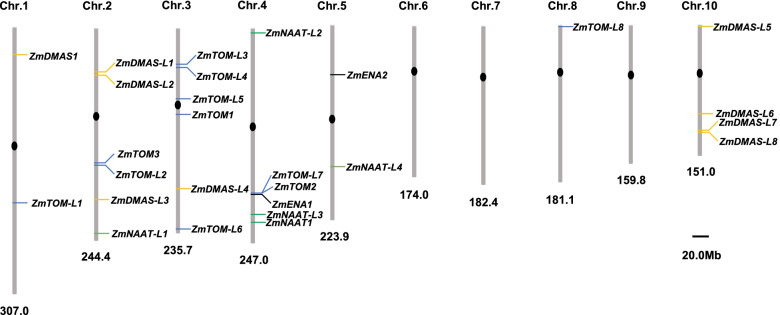
The chromosomal locations of *ZmNAAT*, *ZmDMAS*, *ZmTOM*, and *ZmENA* genes in maize. The positions of identified genes were mapped on the maize genome. The length of the chromosome is indicated at the bottom of each chromosome. *ZmNAAT*, *ZmDMAS*, *ZmTOM*, and *ZmENA* genes are indicated in different colors

The *ZmNAAT*, *ZmDMAS*, *ZmTOM*, and *ZmENA* genes were mapped on the maize genome (Fig. 1). We found that 22 out of 27 genes mapped to chromosomes 2, 3, 4, and 10, while the remaining genes mapped to chromosomes 1, 5, and 8. We also found several closely localized gene pairs, including *ZmDMAS-L1*/*ZmDMAS-L2*, *ZmDMAS-L7*/*ZmDMAS-L8*, *ZmTOM3*/*ZmTOM-L2*, *ZmTOM-L3*/*ZmTOM-L4*, and *ZmTOM-L7*/*ZmTOM2/ZmENA1*.

### Gene structure, phylogenetic analysis, and domain conservation

**Fig. 2:**
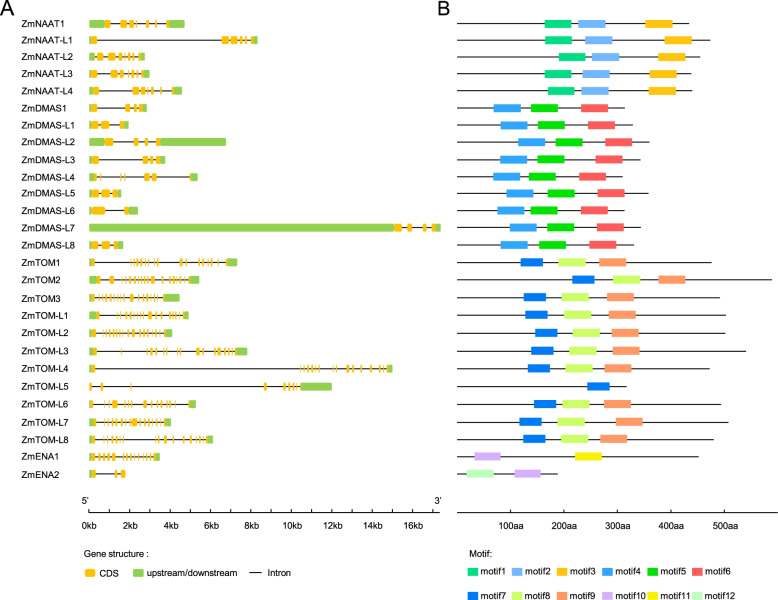
Gene structure and conserved motifs of the *ZmNAAT*, *ZmDMAS*, *ZmTOM*, and *ZmENA* family genes. **A** Yellow boxes indicate the coding sequence (CDS), green blocks indicate untranslated regions, and black lines indicate introns. **B** Motifs of ZmNAAT, ZmDMAS, ZmTOM, and ZmENA proteins. The conserved motifs were analyzed by MEME online software

**Fig. 3:**
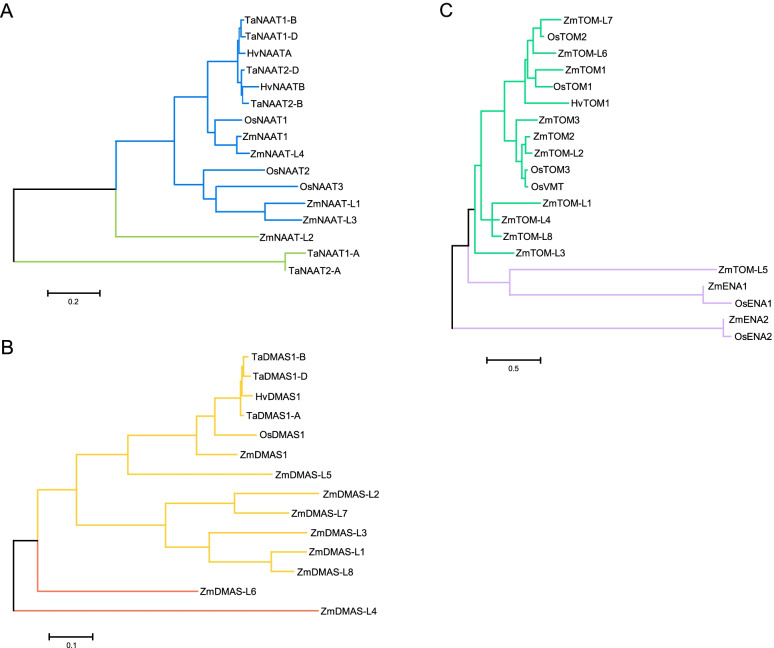
Phylogenetic trees of NAAT, DMAS, TOM, and ENA members from various species. **A** - **C** Phylogenetic tree of (**A**) NAAT proteins, (**B**) DMAS proteins, and (**C**) TOM and ENA proteins. The phylogenetic trees were built with proteins from maize (Zm), wheat (Ta), rice (Os), and barley (Hv) using the maximum likelihood method in MEGA 7.0 software. The proteins and accession numbers used in the phylogenetic trees can be found in the methods. The scale bar corresponds to 10 changes per 100 amino acid positions

We observed conservation in gene structure and protein motifs for ZmNAAT, ZmDMAS, ZmTOM, and ZmENA members (Fig. 2). Amino acid sequence alignment revealed high identities within NAAT and DMAS proteins, while TOM and ENA members exhibited modest similarities (Fig. S1). To investigate their phylogenetic relationships, we constructed phylogenetic trees using ZmNAATs, ZmDMASs, ZmTOMs, ZmENAs, and their homologs in other species (Fig. 3). ZmNAAT proteins were closely related to their rice homologs, as ZmNAAT1 and ZmNAAT-L4 formed a sub-cluster with previously characterized OsNAAT1, while ZmNAAT-L1 and ZmNAAT-L3 formed another sub-cluster with OsNAAT2 and OsNAAT3 (Fig. 3A). This suggests that members within the same sub-cluster may have similar molecular characteristics, such as subcellular localization or gene expression patterns. In addition, we found a close relationship between HvNAATs and TaNAATs, indicating that these genes may have arisen prior to the divergence of barley and wheat (Fig. 3A). The phylogenetic distance between ZmDMAS1 and OsDMAS1 was closer than that between ZmDMAS1 and ZmDMAS-like proteins, suggesting that ZmDMAS-like proteins evolved after the divergence of maize and rice (Fig. 3B). TOMs and ENAs grouped into different clusters in accordance with their different substrate specificity in transporting MAs and NA, respectively (Fig. 3C).

### Expression profiles of *ZmNAAT*, *ZmDMAS*, *ZmTOM*, and *ZmENA* genes under iron-excess and iron-deficient conditions

**Fig. 4:**
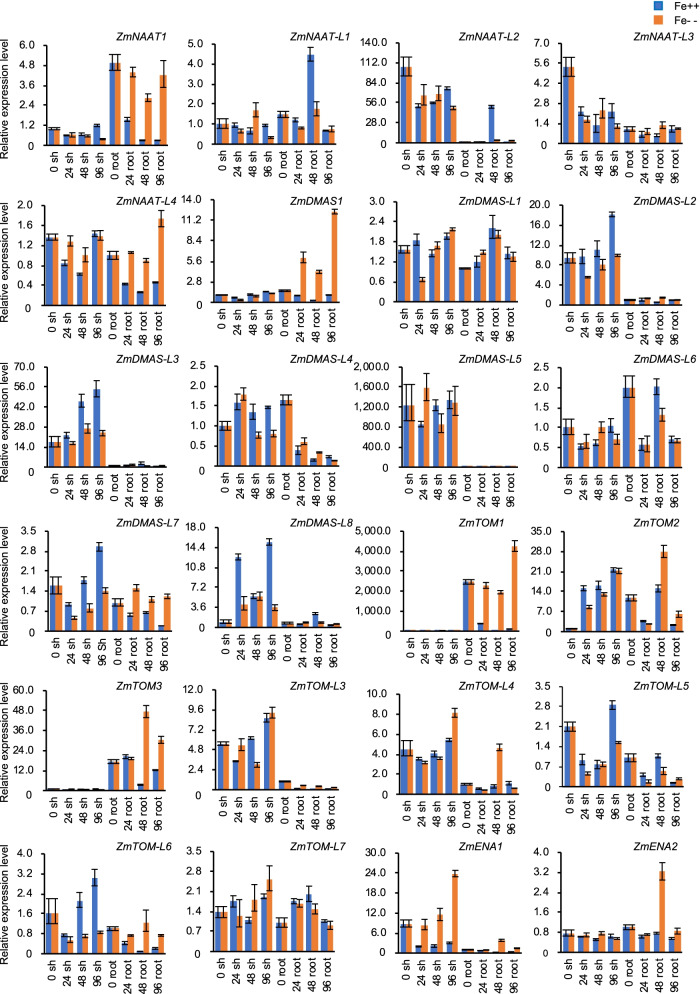
Expression profiles of *ZmNAAT, ZmDMAS*, *ZmTOM*, and *ZmENA* genes in response to different iron levels. The maize seedlings were cultured to the three-leaf stage in standard Hoagland solution and then transferred to Hoagland solution with 0 or 500 μM Fe for deficiency (Fe--) and Fe excess (Fe++) treatments, respectively. The shoots (sh) and roots (root) were harvested at 0, 24, 48, and 96 h after treatments. Maize *Actin1* was used to normalize relative gene expression. The error bars indicate standard deviations

The biosynthesis and secretion of phytosiderophores is essential for iron acquisition in roots, while NA, DMA, and MAs are important for chelating iron in phloem tissues, which indicates their involvement in iron transport and detoxification in vegetative tissues. Therefore, we examined the response of *ZmNAAT*, *ZmDMAS*, *ZmTOM*, and *ZmENA* genes under different iron conditions using quantitative reverse-transcription PCR (qRT-PCR). The expression of *ZmNAAT* genes, encoding enzymes regulating the first step of DMA biosynthesis, exhibited two types of patterns. *ZmNAAT1* and *ZmNAAT-L4* expression was significantly reduced in roots under iron-excess conditions. *ZmNAAT-L2* and *ZmNAAT-L3* expression trended downward in iron-deficient and iron-excess conditions in shoots (Fig. 4). Therefore, *ZmNAAT* genes encoding proteins in different phylogenetic sub-clusters tended to have different expression profiles, as ZmNAAT1/ZmNAAT-L4 and ZmNAAT-L2/ZmNAAT-L3 belong to different sub-clades.

The expression of *ZmDMAS* genes also differed in response to fluctuating iron status. In iron-starved root tissues, *ZmDMAS1* expression was stimulated, while *ZmDMAS-L4* and *ZmDMAS-L*6 were repressed (Fig. 4). In iron-excess shoot samples, *ZmDMAS-L2*, *ZmDMAS-L3*, *ZmDMAS-L7*, and *ZmDMAS-L8* expression was induced (Fig. 4). The different expression patterns of *ZmDMAS* genes also matched their phylogenetic divergence. The up-regulation of *ZmDMAS* genes in shoots grown at high iron concentrations may reflect the demand for DMA biosynthesis, which facilities iron transport and detoxification.

*ZmTOM1* is the causal gene for the *ys3* mutant [26]. We found *ZmTOM1* and *ZmTOM3* expression was significantly induced in iron-deficient roots, suggesting their function in MA secretion in the root. In iron-starved shoots, *ZmTOM-L7* expression increased, while that of *ZmTOM-L5* was reduced. Moreover, *ZmTOM2* was stimulated in iron-deficient and iron-excess shoots (Fig. 4). The up-regulation of *ZmTOM* genes observed in shoots indicated they may be involved in iron transport. Unlike ZmTOM1, the physiological function of ZmENA1 has not been reported. *ZmENA1* expression was increased in response to iron starvation, while it was repressed by excess iron (Fig. 4), indicating a function of *ZmENA1* in iron uptake and transport.

The E-box (CANNTG) and G-box (CACGTG) are *cis*-elements associated with gene expression regulation under fluctuating environmental iron conditions [33, 34]. Both the E-box and G-box are recognized by basic Helix-Loop-Helix (bHLH) family transcription factors, and several bHLHs are regulators of iron homeostasis, e.g., AtbHLH34, AtbHLH38, AtbHLH39, AtbHLH104, AtbHLH115, and OsbHLH156 [34–39]. Thus, we analyzed the 2000-bp promoter region upstream of the start codon of *ZmNAAT*, *ZmDMAS*, *ZmTOM*, and *ZmENA* genes for the presence of E-boxes and G-boxes using the Plant *cis*-Acting Regulatory DNA Elements (PLACE) database [40]. We found the E-boxes and G-boxes were ubiquitous in all analyzed genes (Fig. S2), indicating that bHLH transcription factors may regulate their expression in response to iron status.

### Gene expression profiles in different tissues

**Fig. 5:**
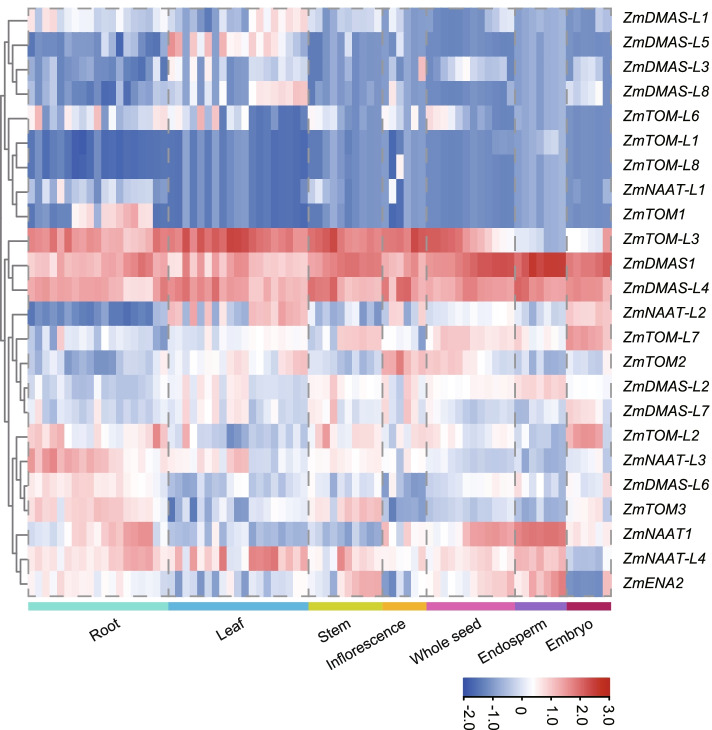
Heatmap showing the expression of *ZmNAAT*, *ZmDMAS*, *ZmTOM*, and *ZmENA* genes in different tissues and developmental stages. The heatmap was generated using RNA-seq data of 79 samples covering the whole lifespan of maize. Color scale represents expression intensity

**Fig. 6:**
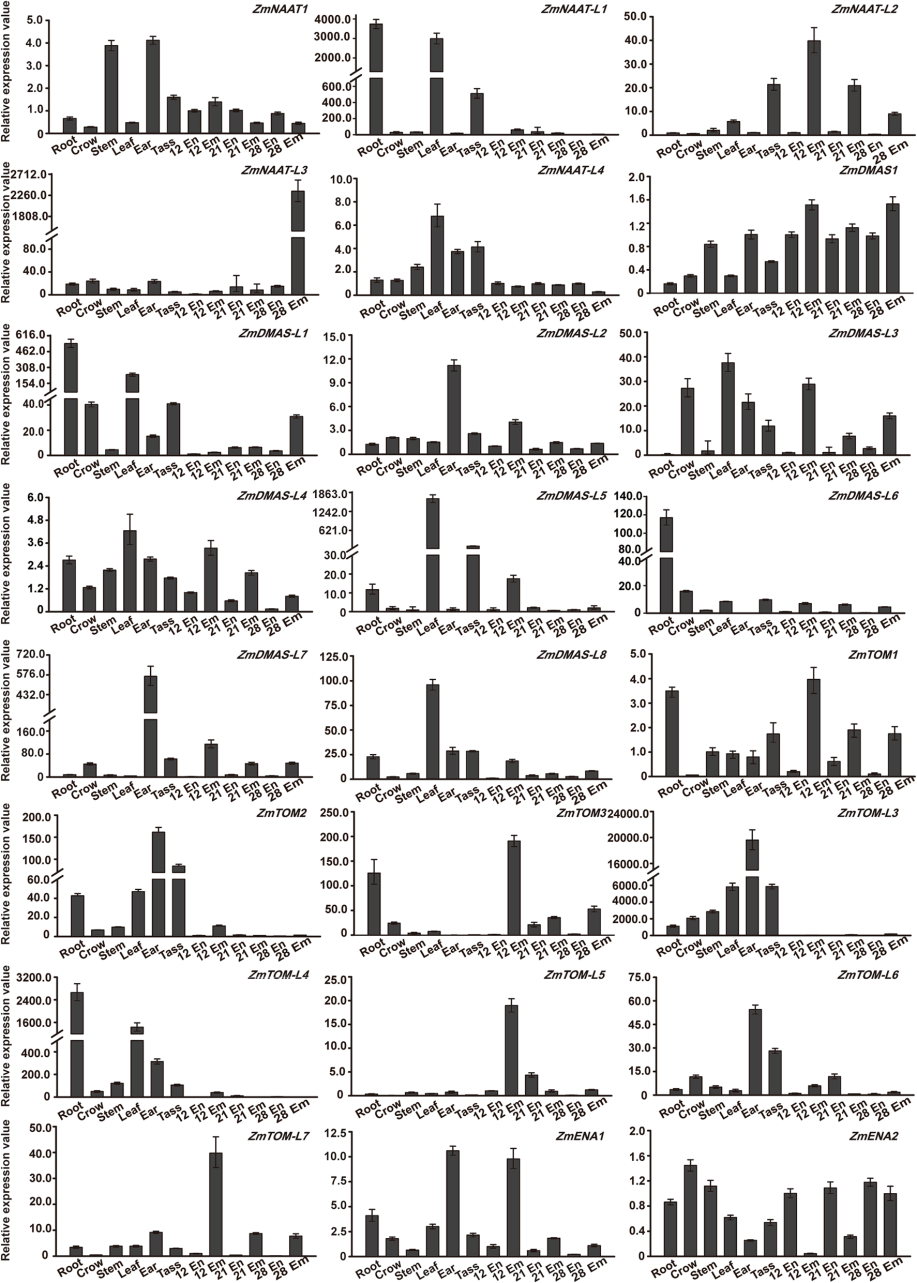
Expression profiles of *ZmNAAT*, *ZmDMAS*, *ZmTOM*, and *ZmENA* genes in different organs and developing seeds. Total RNA was extracted from the endosperm (En) and embryo (Em) at 12, 21, and 28 days after pollination (DAP), as well as from different organs including root (Root), crown root (Crow), stem (Stem), leaf (Leaf), ear (Ear), and tassel (Tass). Maize *Actin1* was used to normalize the relative expression of each gene. The error bars indicate standard deviations

We analyzed the expression of *ZmNAAT*, *ZmDMAS*, *ZmTOM*, and *ZmENA* genes in different tissues using RNA-seq data (Fig. 5) and qRT-PCR (Fig. 6). The heatmap was generated using normalized expression values of 79 samples covering the whole lifespan of maize. Accordingly, we classified the expression pattern of genes associated with phytosiderophore biosynthesis and secretion into five groups, including leaf-preferred (*ZmDMAS-L1/L2/L3/L4/L5/L8*, *ZmNAAT-L2/L4*, and *ZmTOM2/L3*), embryo-preferred (*ZmTOM-L*7 and *ZmDMAS-L7*), root-preferred (*ZmTOM-1/3/L2/L6*, *ZmDMAS1/L6*, *ZmNAAT1/L1/L3*, and *ZmENA2*), endosperm-specific (*ZmTOM-L1*), and anther-specific (*ZmTOM-L8*). Previously identified *ZmNAAT1*, *ZmDMAS1*, and *ZmTOM1* exhibited root-preferential expression patterns, suggesting they may work together in phytosiderophore biosynthesis and secretion in root cells. Newly identified genes were expressed in almost all tissues with different specificities, implying essential roles of phytosiderophores in iron trafficking and storage.

Since expression values of some genes were not available in the RNA-seq data, we used qRT-PCR to determine and further verify their expression in different tissues (root, crown root, stem, leaf, ear, and tassel during flowering and embryo and endosperm development). We found some genes exhibited abundant expression in the ear and/or tassel, including *ZmNAAT1/L2/L4*, *ZmDMAS1/L2/L3/L4/L7*, and *ZmTOM1/3/L5/L7* (Fig. 6). This suggests that phytosiderophore-mediated iron trafficking is involved in reproductive development.

We also observed altered expression specificities via our qRT-PCR analysis, as some leaf- and root-preferential genes showed expression in the stem, tassel, ear, and developing seeds, possibly due to the different sets of tissues and developmental stages used in our study compared with previous studies. *ZmTOM-L4* was preferentially expressed in the leaf and root, while *ZmTOM-L5* expression was mainly detected in the embryo at 12 days after pollination (DAP). *ZmENA1* was expressed in almost all tissues with relatively high expression in the ear and embryo at 12 DAP (Fig. 6).

### Subcellular localization

**Fig. 7:**
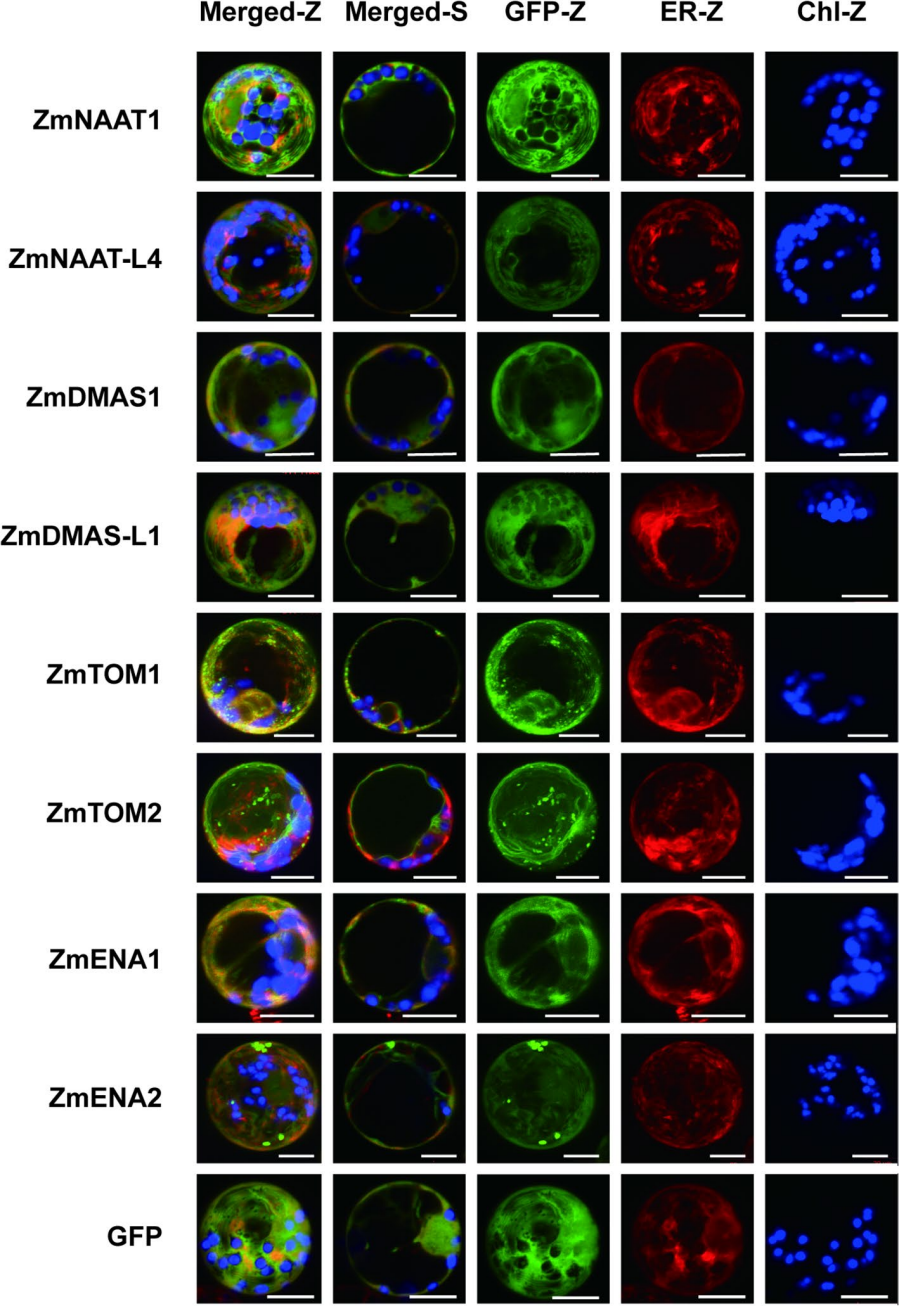
Subcellular localization of ZmNAAT, ZmDMAS, ZmTOM, and ZmENA proteins in maize mesophyll protoplasts. GFP was fused to the C terminus of each gene, and the fusion proteins were co-expressed with an mCherry-fused endoplasmic reticulum (ER) marker in maize mesophyll protoplasts. The GFP signal is shown in green, the ER marker is shown in red, and chlorophyll autofluorescence (Chl) is shown in blue. The images were obtained by confocal microscopy. Z-stacked (Z) and single optical (S) slides of the merged channels are shown. The cytoplasmic localization of GFP was used as a control. The scale bar represents 10 μm

The subcellular localization of a protein indicates its function. We fused randomly selected isoforms of ZmNAAT, ZmDMAS, ZmTOM, and ZmENA proteins with green fluorescent protein (GFP) and transiently expressed them in maize mesophyll protoplasts. All the selected ZmNAAT and ZmDMAS proteins (ZmNAAT1, ZmNAAT-L4, ZmDMAS1, and ZmDMAS-L1) localized in the cytoplasm and nucleus, as did the GFP control (Fig. 7).

ZmTOM and ZmENA proteins localized at membrane systems. Since plasma membrane and complex endomembrane localization was observed for ZmTOM1, ZmENA1, and ZmENA2, we co-transformed an endoplasmic reticulum (ER) marker to specify the inner membrane localization. Because the GFP signals are not fully overlapped with the ER marker in both single and z-stacked images, we speculate that the inner membrane localizations are not the ER. We found spot-like signals for ZmTOM1 and ZmENA2, suggesting they are targeted to small vesicles. ZmTOM2 localized to both the tonoplast and spot-like vesicles, indicating it may mediate import-flux of phytosiderophores into the vacuole.

## Discussion

Graminaceous plants use a chelation strategy to take up sufficient iron and prevent its overaccumulation, and the balance of NA-MA metabolism plays a crucial role in regulating these processes. DMA and MAs are secreted into the rhizosphere to acquire Fe^3+^, while NA and DMA are essential for intercellular, phloem, and possibly xylem iron transport. Since NA also serves as the intermediate for DMA/MA biosynthesis, the export of NA (mediated by ENA) and conversion of DMA (mediated by NAAT and DMAS) are key steps affecting iron homeostasis in plants. Over-consumption of NA induced by constitutively expressing *HvNAAT* in transgenic tobacco plants leads to interveinal chlorosis in young leaves [27]. Similarly, despite the high content of iron in leaves and roots, the lack of NA results in leaf chlorosis in the tomato (*Solanum lycopersicum*) mutant *chloronerva* [41]. These results indicate that NA mediates not only long-distance metal transfer but also metal transport between cells. Disrupted phytosiderophore secretion also generates yellow stripe phenotypes, as defective MA release was identified in the maize *ZmTOM1* mutant (*ys3*) [24, 25]. Therefore, disturbed metabolism and transport of NA/DMA may lead to inadequate iron uptake and distribution.

*NAS* genes are encoded by multi-gene families in a broad range of graminaceous plants, including maize, rice, wheat, and barley [32, 42–44]. *NAS* genes are grouped into class I and class II [32, 44]. Class I *NAS* genes are preferentially expressed in root and stem tissues, and their expression is up-regulated in response to iron deficiency. Conversely, class II genes are mainly expressed in the leaf and are up-regulated in response to excess-iron conditions [32, 45]. In line with these observations, a recent expression and functional study of *OsNAS3* suggests that NA biosynthesized by OsNAS3 under excess-iron conditions is associated with iron detoxification, redistribution, and storage, while NA produced by OsNAS1 and OsNAS2 under normal iron conditions may play roles in iron transport [46]. These results indicate that the expression of the two classes of *NAS* genes may be differentially regulated under fluctuating iron levels, and they may associate with different transporters or enzymes and thereby participate in NA secretion or DMA biosynthesis to balance iron uptake, transport, and storage. This raises the question of whether enzymes associated with NA-DMA metabolism (NAAT and DMAS) and transport for NA/DMA secretion (ENA and TOM) are also encoded by multi-gene families, which could allow for fine regulation of these pathways.

*NAAT* and *DMAS* genes were duplicated in wheat. Six *NAATs* were identified in rice, but only *OsNAAT1* responded to fluctuations in iron levels [47]. Here, in addition to the previously reported *ZmNAAT1*, *ZmDMAS1*, and *ZmTOM1/2/3*, we identified four *ZmNAATs*, eight *ZmDMASs*, eight *ZmTOMs*, and two *ZmENAs* in maize. The expression of *ZmNAAT* and *ZmDMAS* genes was regulated differently in response to environmental iron conditions, though the expression patterns were consistent with their phylogenetic classifications. *ZmNAAT1* and *ZmNAAT-L4* belonged to the same sub-class and were repressed in roots under iron-excess conditions, whereas *ZmNAAT-L2* and *ZmNAAT-L3* were grouped into another sub-class and exhibited reduced expression in iron-deficient and iron-excess conditions in shoots. Likewise, three different expression patterns were observed for *ZmDMAS* genes. In response to iron deficiency, *ZmDMAS1* showed a similar expression trend in roots with that of its orthologs *OsDMAS1*, *HvDMAS1*, and *TaDMAS1* [11]. The close phylogenetic relationship and similar expression trends under fluctuating iron conditions indicate that ZmDMAS1 and OsDMAS1 have a conserved function, as both ZmDMAS1 and OsDMAS1 showed DMA biosynthesis activities in vitro [11]. The physiological function of OsDMAS1 was further confirmed in knock-down plants [48]. In contrast to the increased expression of *ZmDMAS1* under iron deficiency, the expression of *ZmDMAS-L2*, *ZmDMAS-L3*, *ZmDMAS-L7*, and *ZmDMAS-L8* was stimulated in shoots in iron-excess conditions. In addition, *ZmDMAS-L4* and *ZmDMAS-L*6 expression was repressed in roots under iron deficiency. The expression trends of these *DMAS* family genes were associated with their classifications, indicating their potential roles in balancing iron uptake and homeostasis.

Although TOMs and ENAs belong to the MFS family, their amino acid sequences were less conserved compared with DMAS proteins, which may suggest that these TOMs and ENAs have different substrate specificities. ENA is an NA efflux transporter, and *OsENA1* expression was strongly up-regulated under iron-deficient conditions [8, 29]. Similarly, induced expression in response to iron starvation was also observed for *ZmENA1*. As a member of another sub-class of the MFS family, TOM was first identified as a MA efflux transporter in rice and barley, and *OsTOM1/2* and *HvTOM1* expression is induced in iron-deficient roots [8, 24]. Although only three *TOM* and two *ENA* genes were identified in rice, they had a broad spectrum of expression patterns. *OsTOM1* was expressed in shoots and roots, while *OsTOM2* was expressed in the epithelium, scutellum, and dorsal vascular bundles of seeds [8, 24]. *OsENA1* expression was stimulated under iron deficiency [29]. Here, we found that *ZmENA1* expression was induced and repressed in shoots under iron deficiency and excess, respectively. We found that the *TOM* gene family had expanded in maize, and the expression of these *ZmTOM* genes exhibited diverse tissue specificities and different responses to environmental iron conditions. Even though most *ZmTOM* genes were leaf- and root-preferentially expressed, embryo- (*ZmTOM-L5/L7*), endosperm- (*ZmTOM-L1*), and anther-preferred (*ZmTOM-L8*) expression patterns were also identified. *ZmTOM1* and *ZmTOM3* expression was induced by iron deficiency, while that of *ZmTOM2* was stimulated under iron-excess and -deficient conditions. Therefore, we speculated that duplication of *ZmTOM* genes may result from the need for fine regulation of DMA/MA secretion in maize.

The subcellular compartmentalization of enzymes and transporters provides another layer of functional regulation. NA and DMA are biosynthesized in specific vesicles, which may be derived from the ER [49, 50]. Furthermore, OsNAS2 localized in moving vesicles, depending on its tyrosine (YXXφ) and di-leucine (LL) motifs [51]. These vesicles are likely the site of NA and MA biosynthesis and provide a compartment to sequester MAs from the cytosol to maintain iron homeostasis. However, since these vesicles cannot fuse directly with the cell membrane, unidentified transporters might be needed to transport NA/MAs from vesicles into the cytoplasm [51]. We previously revealed that, in contrast to the vesicle-localized OsNASs, ZmNAS proteins are distributed uniformly in the cytoplasm of mesophyll protoplasts [32]. In line with this observation, all the examined ZmNAATs and ZmDMASs showed cytoplasmic localization, suggesting that NA and DMA biosynthesis occurs in the cytoplasm in maize. ZmTOM1, ZmENA1, and ZmENA2 localized at the plasma membrane and endomembrane, and spot-like signals were also observed for ZmTOM1 and ZmENA2. Similarly, OsTOM1 and OsTOM2 were localized on the plasma membrane [8, 24], while OsENA1 localized mainly to the plasma membrane and partially to vesicular structures in rice roots [29]. This result indicates that OsENA1 is likely to be responsible for NA trafficking between the cell membrane and cellular compartments by vesicular transport. Therefore, we speculate that the cytosolic NA and MAs might be exported into vesicles and out of the cell membrane by ZmENA and ZmTOM family proteins. Since the vesicles were proposed to be derived from the ER [51], we applied an ER-marker to determine the nature of the endomembrane. Unexpectedly, the endomembrane localization of ZmTOM1, ZmENA1, and ZmENA2 did not appear to be the ER, though they may interact with or be related to the ER as we found closely related but not identical subcellular localization patterns for GFP and ER-mCherry signals. The vacuole, chloroplast, and mitochondria contain cellular iron pools, and the mobilization of iron between the cytoplasm and these organelles plays essential roles in maintaining iron homeostasis [52–54]. ZINC-INDUCED FACILITATOR1 (ZIF1) is a vacuolar membrane MFS family protein that was hypothesized to transport NA from the cytoplasm into vacuoles [55]. Here, ZmTOM2 targeted to the tonoplast and vesicles, suggesting that ZmTOM2 may act in both vacuolar and vesicular transport of MAs. In summary, our results indicate that ZmTOMs and ZmENAs may contribute to not only NA/MA export into the intercellular space but also to NA/MA sequestration into the vacuole and vesicles to detoxify excess iron.

One way to improve essential micronutrients in the human diet is by enhancing the iron content in cereal grains. Several approaches have been used to enhance the iron content in seeds, including upregulation of genes associated with iron uptake and transport, as well as modification of the expression of endosperm-specific genes [53, 56]. It is noteworthy that excessive production of NA leads to an increase in DMA, which may increase the iron content in seeds [28]. Consistent with this idea, different NA-to-DMA ratios regulated by NAS and NAAT had different effects on the iron content in grains [28, 57]. Therefore, further exploration of enzymes involved in the biosynthesis and transport of NA and DMA may provide a theoretical basis to optimize iron biofortification in cereals.

## Conclusions

In this study, we identified 5 *ZmNAAT*, 9 *ZmDMA*, 11 *ZmTOM*, and 2 *ZmENA* genes in maize and determined their phylogenetic relationships, subcellular localization, and gene expression patterns in different tissues and under different iron conditions. We found abundant expression of these genes in the tassel, ear, and developing seeds, indicating essential roles of NA and phytosiderophores in iron transport during reproductive development. In addition, the expression of these genes was regulated by iron status, suggesting that the balance between NA and phytosiderophores within plant cells is essential for iron homeostasis. All the examined ZmNAATs and ZmDMASs localized in the cytoplasm, whereas ZmTOMs and ZmENAs localized in the plasma and tonoplast membrane, endomembrane, and vesicle. This indicates that ZmTOMs and ZmENAs may contribute to intercellular export and intracellular sequestration of NA and phytosiderophores. Furthermore, the expansion of the *ZmNAAT*, *ZmDMA*, *ZmTOM*, and *ZmENA* gene families suggests that fine regulation of NA-phytosiderophore metabolism contributes to iron homeostasis in maize. These results provide valuable insight into the potential functions of *ZmNAAT*, *ZmDMA*, *ZmTOM*, and *ZmENA* genes for breeding iron-rich maize varieties.

## Methods

### Plant materials and growth conditions

The maize (*Zea mays* L.) inbred line Z58 was obtained from the Center for Crop Germplasm Resources, Chinese Academy of Agricultural Sciences. All seeds were planted in a greenhouse. For expression analysis in different tissues, we collected root, crown root, stem, leaf, ear, and tassel tissues before pollination. We also collected embryo and endosperm at 12, 21, and 28 days after pollination (DAP) to determine expression patterns in developing seeds. For the different iron treatments, Z58 seedlings were cultured at 28 °C with a 16-h-light/8-h-dark cycle. The seeds were germinated, and the seedlings were incubated in normal Hoagland nutrient solution with Fe^3+^-EDTA (Fe^3+^ concentration = 100 μM) until the trefoil stage. Then, the seedlings were transferred to Hoagland nutrient solution without Fe^3+^-EDTA or with excessive Fe^3+^-EDTA (Fe^3+^ concentration = 500 μM) for deficient and excess iron treatments, respectively. Shoots and roots were harvested at 24, 48, and 96 h after the different Fe^3+^-EDTA treatments. Seedlings sampled before the iron treatments were used as controls (0 h). All samples were immediately frozen in liquid nitrogen and quickly stored at − 80 °C.

### Bioinformatics analysis

To identify the *NAAT*, *DMAS*, *TOM*, and *ENA* family genes in maize, we used the previously characterized members of these families in rice (*Oryza sativa* L.) and maize as queries (Table S1). First, the TBLASTN and BLASTP program at Gramene (http://ensembl.gramene.org/Zea_mays/Tools/Blast) was used to identify putative homologous proteins. Candidates were obtained with a set of relatively sensitive cutoffs (Table S3) to remove low degrees of similarity and inferior domain coverage, as well as proteins annotated with different functions.

Next, we used HMMER to test the putative proteins. The HMM for the canonical domains of NAAT (PF00155), DMAS (PF00248), and TOM/ENA (PF07690) were obtained from the Pfam database (http://pfam.xfam.org/) and used as queries against the maize annotated protein database using HMMER. Table S3 shows the cutoffs used to avoid false positives. The ExPASy ProtParam tool (https://web.expasy.org/protparam/) was used to calculate the physicochemical properties of each protein, including the number of amino acids (aa), isoelectric point (pI), and molecular weight (MW). WoLF POSRT II (https://wolfpsort.hgc.jp/) and Plant-mPLoc (http://www.csbio.sjtu.edu.cn/bioinf/plant-multi/) were used to predict the subcellular locations of each protein. All genes were mapped to maize chromosomes and named according to their locations. The gene structures were analyzed in GSDS v.2.0. The MEME online software (https://meme-suite.org/meme/) was used to predict the conserved domain of proteins. For *cis*-element analysis, 2000-bp upstream promoter sequences from the translation start site were analyzed in the PLACE database (https://www.dna.affrc.go.jp/PLACE/?action=newplace), and the G-box and E-box distribution was visualized by TBtools software [58]. The phylogenetic tree was constructed for NAAT, DMAS, TOM, and ENA proteins from different species using the Maximum Likelihood method in MEGA version 7.0 (Pennsylvania State University, State College, PA, USA) with the bootstrap method of 1000 replicates. The amino acid sequences encoded by selected genes were compared with known genes by the MegAlign software of Lasergene using the ClustalW method, and the alignments were visualized by GeneDoc software.

### Expression analysis using RNA-seq data

The expression values of all selected genes in various tissues were retrieved from previous RNA-seq data of 79 tissues covering the whole lifespan of maize [59]. The heatmap was construed and visualized using TBtools software [58].

### Quantitative reverse-transcription PCR

Total RNA was extracted using a Plant RNA Kit (Transgen, Beijing, China) following the manufacturer’s instructions. The first-strand cDNA was obtained by reverse transcription using cDNA TranScript One-Step gDNA Removal and cDNA Synthesis SuperMix (Transgen, Beijing, China). Primers for qRT-PCR assays (Table S4) were designed using the Primer 3.0 website (http://bioinfo.ut.ee/primer3-0.4.0/). qRT-PCR was performed in a 20-μL volume containing 10 μL SYBR Green (Takara, Japan), 0.4 μL ROX II (Takara, Japan), 0.2 μM gene-specific primers, and 5 μL 5× diluted cDNA. The reaction was performed using the ABI 7500 Real Time Thermal Cycler. The expression of *ZmActin1* was used as an internal control (Table S4). For qRT-PCR assays, three biological replicates were performed, with three technical replicates per biological replicate.

### Subcellular localization

To construct plant transient expression vectors, the amplified open reading frames (ORFs) were cloned into the XhoI-XbaI site of the pRTL-2NGFP plasmid [32] to express C-terminal GFP fusion proteins. Gene-specific primers were designed to amplify the full-length ORFs without stop codons (Table S4). To determine the subcellular localization of randomly selected NAAT, DMAS, ENA, and TOM family proteins, the GFP-fusion proteins were co-expressed in maize mesophyll protoplasts with an ER marker, a chimeric protein generated by combining the signal peptide of AtWAK2 (*Arabidopsis thaliana* WALL-ASSOCIATED KINASE 2) at the N terminus of mCherry and ER retention signal His-Asp-Glu-Leu at its C terminus [60]. For mesophyll protoplast transformation, plasmids were extracted using the Wizard Plus Miniprep DNA Purification System kit (Promega, Beijing, China). Maize seedlings were grown in a greenhouse in the dark. Protoplasts were extracted and transformed using polyethylene glycol 4000 as described previously [61]. After co-transformation, the protoplasts were incubated in darkness at 26 °C for 12–16 h. Then, a confocal microscope (LSM700; Carl Zeiss) was used to visualize the fluorescence. GFP and mCherry signals were excited at 488 and 555 nm, and their emission was collected at 500–530 nm and 610 nm, respectively. Chlorophyll autofluorescence was observed using the 650-nm emission filter. ZEN light edition 2009 software was used for image processing.

## Supplementary Information

**Additional file 1: Figure S1**. The multiple sequence alignments of NAAT, DMAS, TOM, and ENA proteins.

**Additional file 2: Figure S2**. E-boxes and G-boxes in the *ZmNAAT*, *ZmDMAS*, *ZmTOM*, and *ZmENA* promoter regions.

**Additional file 3: Table S1**. Genes used for the identification and phylogenetic analysis of the NAAT, DMAS, TOM, and ENA families in maize.

**Additional file 4: Table S2**. Identified NAAT, DMAS, TOM, and ENA proteins in maize, wheat, barley, and rice.

**Additional file 5: Table S3**. The cutoffs for the identification of NAAT, DMAS, TOM, and ENA proteins in maize using BLAST and HMMER.

**Additional file 6: Table S4**. Primers used in this study.

## Data Availability

The public transcriptome data sets corresponding to expression analysis can be downloaded from the National Center for Biotechnology Information Sequence Read Archive (PRJNA171684, https://trace.ncbi.nlm.nih.gov/Traces/sra/sra.cgi?study=SRP014652 and SRP010680, https://trace.ncbi.nlm.nih.gov/Traces/sra/?study=SRP010680). All data and materials generated or analyzed during this study are included in this article or are available from the corresponding author on reasonable request.

## Abbreviations

NA: Nicotianamine
DAP: Days after pollination
DMA: Deoxymugineic acid
DMAS: 2′-Deoxymugineic acid synthase
ENA: Efflux transporter of NA
ER: Endoplasmic reticulum
Fe: Iron
Fe^2+^: Ferrous
Fe^3+^: Ferric
GFP: Green fluorescent protein
HMM: Hidden markov model
IRT1: Iron-regulated transporter 1
MAs: Mugineic acid family phytosiderophores
MFS: Major facilitator superfamily
ML: Maximum likelihood
NAAT: Nicotianamine aminotransferase
NAS: Nicotianamine synthase
PS: Phytosiderophores
qRT-PCR: Quantitative reverse transcription polymerase chain reaction
RNA-seq: RNA sequencing
TOM: MAs efflux transporter
YS1: YELLOW STRIPE 1
YSL: YELLOW STRIPE 1-LIKE
