# Supplementary figures and images for: Genome-wide analysis of the NAAT, DMAS, TOM, and ENA gene families in maize suggests their roles in mediating iron homeostasis
Source: BMC Plant Biol. 2022 Jan 17;22:37. doi: 10.1186/s12870-021-03422-7 (PMC8762928; doi:10.1186/s12870-021-03422-7)

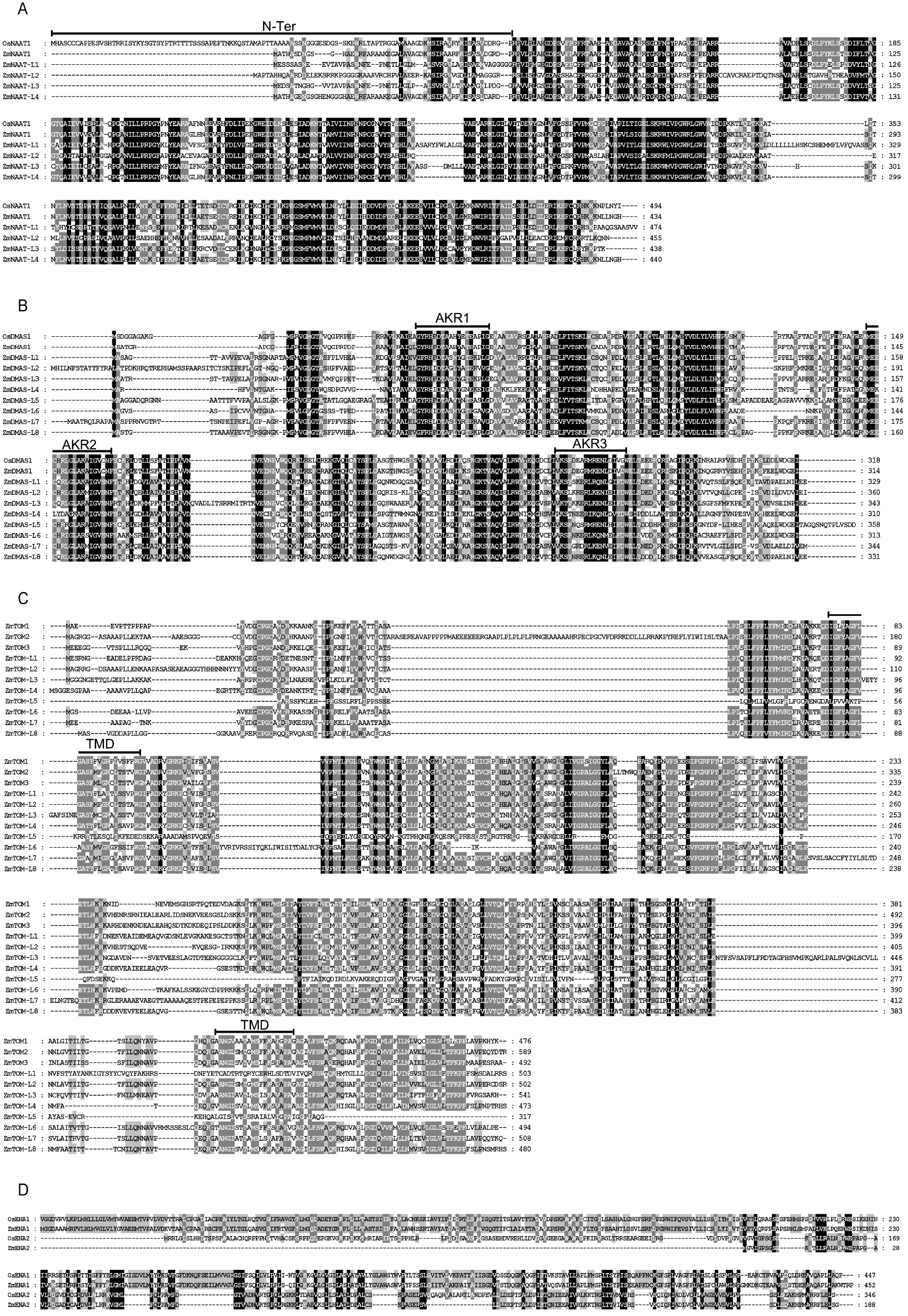

Supplement: Supplementary file 1 — Additional file 1: Figure S1. The multiple sequence alignments of NAAT, DMAS, TOM, and ENA proteins. [file 12870_2021_3422_MOESM1_ESM.jpg]

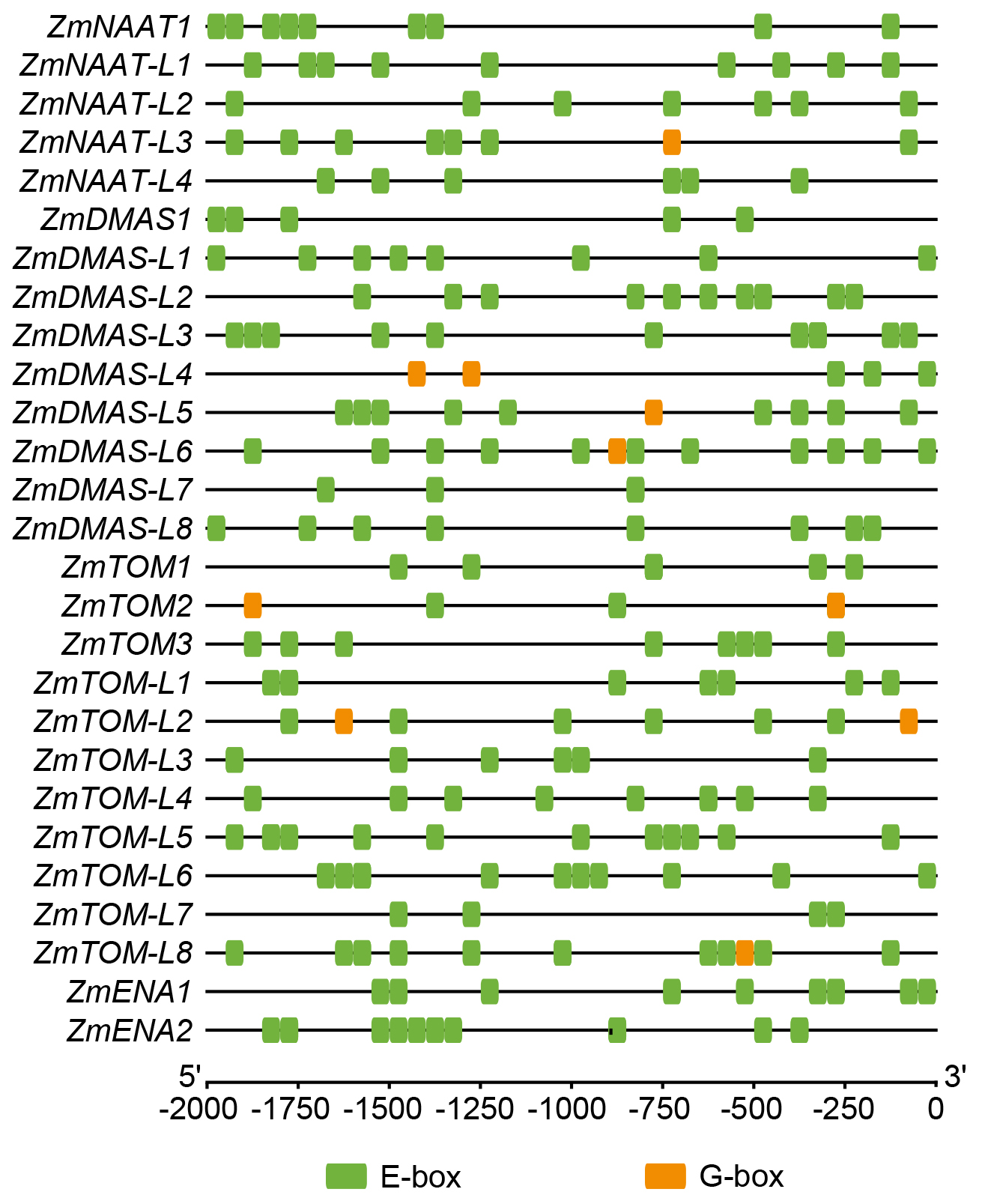

Supplement: Supplementary file 2 — Additional file 2: Figure S2. E-boxes and G-boxes in the ZmNAAT, ZmDMAS, ZmTOM, and ZmENA promoter regions. [file 12870_2021_3422_MOESM2_ESM.jpg]
