# Supplementary material for: Genome-wide analysis of the NAAT, DMAS, TOM, and ENA gene families in maize suggests their roles in mediating iron homeostasis
Source: BMC Plant Biol. 2022 Jan 17;22:37. doi: 10.1186/s12870-021-03422-7 (PMC8762928; doi:10.1186/s12870-021-03422-7)
Supplement: Supplementary file 3 — Additional file 3: Table S1. Genes used for the identification and phylogenetic analysis of the NAAT, DMAS, TOM, and ENA families in maize. [file 12870_2021_3422_MOESM3_ESM.docx]

**Table S1****.** Genes used for the identification and phylogenetic analysis of the NAAT, DMAS, TOM, and ENA families in maize

| Gene name | Gene ID/GenBank accession number |
| --- | --- |
| *ZmNAAT1* | *Zm00001d053281* |
| *OsNAAT1* | *Os02g0306401* |
| *OsNAAT2* | *Os02g0302700* |
| *OsNAAT3* | *Os02g0302400* |
| *TaNAAT1-A* | KX348554 |
| *TaNAAT1-B* | KX348555 |
| *TaNAAT1-D* | KX348556 |
| *TaNAAT2-A* | KX348557 |
| *TaNAAT2-B* | KX348558 |
| *TaNAAT2-D* | KX348559 |
| *HvNAAT-A* | BAA87052 |
| *HvNAAT-B* | BAA87053 |
| *ZmDMAS1* | *Zm00001d028360* |
| *OsDMAS1* | *Os03g0237100* |
| *TaDMAS1-A* | KX348551 |
| *TaDMAS1-B* | KX348552 |
| *TaDMAS1-D* | KX348553 |
| *HvDMAS1* | BAF03162 |
| *ZmTOM1* | *Zm00001d041111* |
| *ZmTOM2* | *Zm00001d052435* |
| *ZmTOM3* | *Zm00001d005001* |
| *OsTOM1* | *Os11t0134900* |
| *OsTOM2* | *Os11g0135000* |
| *OsTOM3* | *Os11g0135900* |
| *OsVMT* | *Os12g0133100* |
| *HvTOM1* | BAL15698 |
| *OsENA1* | *Os11g0151500* |
| *OsENA2* | *Os06g0695800* |
