# Supplementary material for: Genome-wide analysis of the NAAT, DMAS, TOM, and ENA gene families in maize suggests their roles in mediating iron homeostasis
Source: BMC Plant Biol. 2022 Jan 17;22:37. doi: 10.1186/s12870-021-03422-7 (PMC8762928; doi:10.1186/s12870-021-03422-7)
Supplement: Supplementary file 4 — Additional file 4: Table S2. Identified NAAT, DMAS, TOM, and ENA proteins in maize, wheat, barley, and rice. [file 12870_2021_3422_MOESM4_ESM.docx]

**Table S2.** Identified NAAT, DMAS, TOM, and ENA proteins in maize, wheat, barley, and rice

| Species | NAAT | DMAS | TOM | ENA | References |
| --- | --- | --- | --- | --- | --- |
| Maize (*Zea mays* L.) | 5 | 9 | 11 | 2 | This study |
| Wheat (*Triticum aestivum* L.) | 6 | 3 |  |  | [30, 11] |
| Barley (*Hordeum vulgare* L.) | 2 | 1 | 1 |  | [8, 11, 42] |
| Rice (*Oryza sativa* L.) | 3 | 1 | 3 | 2 | [8, 11, 29, 47] |
