# Supplementary material for: Genome-wide analysis of the NAAT, DMAS, TOM, and ENA gene families in maize suggests their roles in mediating iron homeostasis
Source: BMC Plant Biol. 2022 Jan 17;22:37. doi: 10.1186/s12870-021-03422-7 (PMC8762928; doi:10.1186/s12870-021-03422-7)
Supplement: Supplementary file 5 — Additional file 5: Table S3. The cutoffs for the identification of NAAT, DMAS, TOM, and ENA proteins in maize using BLAST and HMMER. [file 12870_2021_3422_MOESM5_ESM.docx]

**Table S3.** The cutoffs for the identification of NAAT, DMAS, TOM, and ENA proteins in maize using BLAST and HMMER

| Family | Blast manner | Blast protein | *E*-value | Score | % Identity |
| --- | --- | --- | --- | --- | --- |
| ZmNAAT | TBLASTN | ZmNAAT1 | E-value<1*10^-5^ | >450 | >50% |
|  |  | OsNAAT1 | E-value<1*10^-5^ | >450 | >50% |
|  | BLASTP | ZmNAAT1 | E-value<1*10^-5^ | >450 | >50% |
|  |  | OsNAAT1 | E-value<1*10^-5^ | >450 | >50% |
|  | PHMMER | ZmNAAT1 | E-value<1*10^-5^ | >350 | null |
|  |  | OsNAAT1 | E-value<1*10^-5^ | >350 | null |
| ZmDMAS | TBLASTN | ZmDMAS1 | E-value<1*10^-5^ | >450 | >45% |
|  |  | OsDMAS1 | E-value<1*10^-5^ | >450 | >45% |
|  | BLASTP | ZmDMAS1 | E-value<1*10^-5^ | >400 | >40% |
|  |  | OsDMAS1 | E-value<1*10^-5^ | >400 | >40% |
|  | PHMMER | ZmDMAS1 | E-value<1*10^-5^ | >250 | null |
|  |  | OsDMAS1 | E-value<1*10^-5^ | >250 | null |
| ZmTOM | TBLASTN | ZmTOM1 | E-value<1*10^-5^ | >385 | >55% |
|  |  | ZmTOM2 | E-value<1*10^-5^ | >340 | >55% |
|  |  | ZmTOM3 | E-value<1*10^-5^ | >400 | >50% |
|  | BLASTP | ZmTOM1 | E-value<1*10^-5^ | >260 | >50% |
|  |  | ZmTOM2 | E-value<1*10^-5^ | >200 | >50% |
|  |  | ZmTOM3 | E-value<1*10^-5^ | >270 | >50% |
|  | PHMMER | ZmTOM1 | E-value<1*10^-5^ | >150 | null |
|  |  | ZmTOM2 | E-value<1*10^-5^ | >150 | null |
|  |  | ZmTOM3 | E-value<1*10^-5^ | >150 | null |
| ZmENA | TBLASTN | OsENA1 | E-value<1*10^-5^ | >800 | >70% |
|  |  | OsENA2 | E-value<1*10^-5^ | >800 | >70% |
|  | BLASTP | OsENA1 | E-value<1*10^-5^ | >800 | >70% |
|  |  | OsENA2 | E-value<1*10^-5^ | >800 | >70% |
|  | PHMMER | OsENA1 | E-value<1*10^-5^ | >300 | null |
|  |  | OsENA2 | E-value<1*10^-5^ | >300 | null |
