# Supplementary material for: Genome-wide analysis of the NAAT, DMAS, TOM, and ENA gene families in maize suggests their roles in mediating iron homeostasis
Source: BMC Plant Biol. 2022 Jan 17;22:37. doi: 10.1186/s12870-021-03422-7 (PMC8762928; doi:10.1186/s12870-021-03422-7)
Supplement: Supplementary file 6 — Additional file 6: Table S4. Primers used in this study. [file 12870_2021_3422_MOESM6_ESM.docx]

**Table S4.** Primers used in this study

| Primer name | Primer sequence |
| --- | --- |
| *ZmNAAT1-*RT-U | GGCCATTAACTGTATCTACGT |
| *ZmNAAT1-*RT-L | TCTGCGTATTCAAGGAAAAC |
| *ZmNAAT-L1-*RT-U | CCTCTGTAGTCTAGTAGTG |
| *ZmNAAT-L1-*RT-L | TTGACCCAAACTTCATTGTT |
| *ZmNAAT-L2-*RT-U | TGGTTCTGTCATTGGGTTGA |
| *ZmNAAT-L2-*RT-L | ATCTCAATCCTGTCGCAAGC |
| *ZmNAAT-L3-*RT-U | AGTCACCAGTTCTATGCAAC |
| *ZmNAAT-L3-RT*-L | TCAAACATGTCATCTTCGAGT |
| *ZmNAAT-L4-*RT-U | TGATGGTCTTGAGAGGCTGA |
| *ZmNAAT-L4-*RT-L | GTGGAAAACGATGGAAGTGG |
| *ZmDMAS1-*RT-U | CGGCGAGATATGATATGAGT |
| *ZmDMAS1-*RT-L | GACATAAAACCACACAAAGC |
| *ZmDMAS-L1-*RT-U | TGTCCAGACTAGTAGCTTGT |
| *ZmDMAS-L1-*RT-L | TGATGCAGTGTATGTGTGG |
| *ZmDMAS-L2-*RT-U | GAATCTCCTGTGTCCTGAAG |
| *ZmDMAS-L2-*RT-L | GTACACCAATTCTTTTGCCC |
| *ZmDMAS-L3-*RT-U | TTCTGGCTTCTCATTCATGT |
| *ZmDMAS-L3-*RT-L | ATTTCTTTAGCGCCTACTCC |
| *ZmDMAS-L4-*RT-U | GATGTGCTGCTGCAGTCTGT |
| *ZmDMAS-L4-*RT-L | ATGATGCCTGGCGAATTAAC |
| *ZmDMAS-L5-*RT-U | TCTCCTTTGTCACGAAGAAG |
| *ZmDMAS-L5-*RT-L | GTAATGTTTTCTCTCGTGTCG |
| *ZmDMAS-L6-*RT-U | CTGCTGTGGGGAAATATCTT |
| *ZmDMAS-L6-*RT-L | CACCCAACCTAGCTAGAATG |
| *ZmDMAS-L7-*RT-U | CGGCTACGTTGAATCAGTTG |
| *ZmDMAS-L7-*RT-L | GAGGTCCGGACGACAAGTAG |
| *ZmDMAS-L8-*RT-U | TTCCACGATGTTTTCCATCC |
| *ZmDMAS-L8-*RT-L | TCGACCGTTCCAAAGAAGTC |
| *ZmTOM1-*RT-L | ACATAATTCCGTTGTATGCCACT |
| *ZmTOM1-*RT-U | AAATGACAGCTGCTCGAACC |
| *ZmTOM2-*RT-L | GCGCTTCTCCTTCACTGGTA |
| *ZmTOM2-*RT-U | CCAGAGCAGTACGATACGAGAT |
| *ZmTOM3-*RT-L | CGATCCTCCCCTTCCATCTC |
| *ZmTOM3-*RT-U | CCAGAGTCCAGAGCAGCAT |
| *ZmTOM-L3-*RT-L | TACCATACGCCGTGTGTTCT |
| *ZmTOM-L3-*RT-U | CTATCCAGGGGCAATCGAAT |
| *ZmTOM-L4-*RT-L | TTCACAGGAACGGGCTTCAT |
| *ZmTOM-L4-*RT-U | CCTGGTGCTGACGTTCAAG |
| *ZmTOM-L5-*RT-L | GTTTGGTGCTCAGTTCAACACT |
| *ZmTOM-L5-*RT-U | CCTAACAGCCCAAAGCATGT |
| *ZmTOM-L6-*RT-L | TGCACTCGATCAACTGTAACT |
| *ZmTOM-L6-*RT-U | TCCACCTTTGAGCCCTTTCT |
| *ZmTOM-L7-*RT-L | CATCTCCCTCGTCTCGCTC |
| *ZmTOM-L7-*RT-U | TCCCCAGCAGTACAAACAGT |
| *ZmENA1-*RT-U | GGACCAGACCATGACTCCAG |
| *ZmENA1-*RT-L | GGCAAGCCCTATGTTGTTGG |
| *ZmENA2-*RT-U | GTTTCTTGGAGCTCAGCGG |
| *ZmENA2-*RT-L | ACACGTCCGATCCTTTCCAT |
| *ZmActin1*-RT-U | ATGTTTCCTGGGATTGCCGAT |
| *ZmActin1*-RT-L | CCAGTTTCGTCATACTCTCCCTTG |
| *ZmNAAT1*-GFP-Fusion-L | CCTTGCTCACCATTCTAGAATGGCCATTAAGCAAATTCT |
| *ZmNAAT-L4*-GFP-Fusion-U | ATTTGGAGAGGACCTCGAGATGGCGACCCACGCCGGC |
| *ZmNAAT-L4*-GFP-Fusion-L | CCTTGCTCACCATTCTAGAATGGCCATTAAGCAAATTCTTCT |
| *ZmDMAS1*-GFP-Fusion-U | ATTTGGAGAGGACCTCGAGATGAGCGCGACCGGGCGAGC |
| *ZmDMAS1*-GFP-Fusion-L | CCTTGCTCACCATTCTAGATATCTCGCCGTCCCATAGCT |
| *ZmDMAS-L1-*GFP-Fusion-U | ATTTGGAGAGGACCTCGAGATGGCATCCGCGGGGACG |
| *ZmDMAS-L1-*GFP-Fusion-L | CCTTGCTCACCATTCTAGATTCCTCTAAAATATTAAGTTCC |
| *ZmTOM1*-GFP-Fusion-U | ATTTGGAGAGGACCTCGAGATGGCTGAGGAGGTGCCAACGA |
| *ZmTOM1*-GFP-Fusion-L | CCTTGCTCACCATTCTAGATTTATAGTGTTTCGGTACTGCT |
| *ZmTOM2*-GFP-Fusion-U | ATTTGGAGAGGACCTCGAGATGGCGGGCCGGGGAGGA |
| *ZmTOM2*-GFP-Fusion-L | CCTTGCTCACCATTCTAGATCTCGTATCGTACTGCTCTG |
| *ZmENA1*-GFP-Fusion-U | ATTTGGAGAGGACCTCGAGATGGGCGGGGATGCGGCG |
| *ZmENA1*-GFP-Fusion-L | CCTTGCTCACCATTCTAGATGGTCTGGTCCATTTTGCCAGT |
| *ZmENA2*-GFP-Fusion-U | ATTTGGAGAGGACCTCGAGATGGGCGTCATCGGCCCC |
| *ZmENA2*-GFP-Fusion-L | CCTTGCTCACCATTCTAGAGCCGCTGAGCTCCAAGAAACG |
